# The Impact of Bilateral Anodal tDCS over Left and Right DLPFC on Executive Functions in Children with ADHD

**DOI:** 10.3390/brainsci12081098

**Published:** 2022-08-18

**Authors:** Mohammad Ali Salehinejad, Younes Vosough, Vahid Nejati

**Affiliations:** 1 Leibniz Research Centre for Working Environment and Human Factors, Department of Psychology and Neurosciences, 44139 Dortmund, Germany; 2 Institute for Cognitive and Brain Sciences, Shahid Beheshti University, Tehran 1983969411, Iran; 3 Department of Psychology, Shahid Beheshti University, Tehran 1983969411, Iran

**Keywords:** transcranial direct current stimulation, neurodevelopmental disorders, ADHD, DLPFC, tDCS, executive functions

## Abstract

Transcranial direct current stimulation (tDCS) is increasingly used for therapeutic purposes in attention-deficit hyperactivity disorder (ADHD). The dorsolateral prefrontal cortex (DLPFC) is the most targeted region of tDCS studies in ADHD. There is limited knowledge and mixed results about the relevance of left or right DLPFCs in ADHD’s cognitive deficits. No study so far has investigated the impact of the increased excitability of both left and right DLPFC with anodal tDCS on cognitive deficits in ADHD. Here, we explored the impact of online bilateral anodal left and right DLPFC tDCS on executive dysfunction in children with ADHD. Twenty-two children with ADHD (mean age ± SD =8.86 ± 1.80) received bilateral anodal online tDCS over the left and right DLPFC (1.5 mA, 15 min) in two separate sessions in active and sham states. They underwent a battery of four neuropsychological tasks of executive functions during stimulation that measured working memory, cognitive flexibility, response inhibition, and executive control. Bilateral anodal left and right DLPFC tDCS did not improve performance on working memory, cognitive flexibility, and response inhibition. Executive control was, however, partially improved for those who received active tDCS first. The upregulation of bilateral DLPFC tDCS with anodal polarity does not improve executive dysfunction in children with ADHD. The unilateral modulation of DLPFC with anodal tDCS may be more beneficial to cognitive deficits in ADHD in light of previous works targeting only left and/or right DLPFC.

## 1. Introduction

Attention-deficit hyperactivity disorder (ADHD) is the most common neurodevelopmental disorder and is characterized by significant cognitive deficits and/or impulsive behavior, different subtypes, and maladaptive lifestyle behaviors [1,2,3,4]. A well-known and accepted theory of ADHD pathophysiology posits that ADHD is a disorder of executive dysfunction, specifically inhibitory control, due to pervasive cognitive deficits in ADHD [5,6]. Recent advances in brain imaging have shown that ADHD etiology largely comes from abnormalities in the brain structure and function, especially in the frontal-striatal circuitry including the prefrontal regions and related brain networks (e.g., central executive network) [7,8,9] which are related to deficits in both cold (e.g., response inhibition, working memory, sustained attention) and hot (e.g., reward processing, delay discounting) executive functions in ADHD [7,10].

These large brain abnormalities have motivated researchers and clinicians to use safe interventions that can modulate brain functional abnormalities. Transcranial direct current stimulation (tDCS) is a non-invasive brain stimulation technique used for studying and modifying brain functions [11,12,13]. tDCS has been widely used for studying and improving cognitive functions and their underlying brain physiology [14,15,16,17]. Among the target groups in child and adolescent psychiatry, tDCS has been mostly applied to ADHD [18,19,20]. Different but few brain regions have been targeted in tDCS studies conducted on ADHD [21,22,23]. Two brain regions were targeted in the majority of tDCS studies in ADHD: the lateral prefrontal cortex and the r-IFG [20,24]. The DLPFC, specifically the left DLPFC, has been the most often targeted region with anodal stimulation, which is not surprising due to its documented role in executive functions [7,25,26]. The right DLPFC, however, is not directly targeted in ADHD tDCS studies in children, despite its potential role in ADHD pathophysiology. The only tDCS study that specifically investigated the role of the right DLPFC found a partial improving effect of right DLPFC anodal tDCS (single session) in response inhibition that was dependent on symptom severity [27]. Another recently published work in adult ADHD showed improving effects of multisession anodal right cathodal left prefrontal tDCS on attentional deficits [28]. Yet with the current literature, the comparative contributions of the left and right DLPFCs in ADHD are not well-known.

One of the unaddressed questions here is studying the impact of applying anodal tDCS over both left and right DLPFC. Addressing this question is important for several reasons: First, there is no study about the application of bilateral DLPFC anodal tDCS in ADHD. There is only one study that applied slow oscillating tDCS at a frequency of 0.75 Hz over bilateral DLPFC during slow-wave sleep in children with ADHD with anodal polarity and found an improving effect on memory [29]. This is also a relevant question because the neurobiological model of executive functions assumes that both left and right DLPFCs are involved in core executive functions such as working memory [26] and previous tDCS studies show that working memory and response inhibition are related to the activity of both DLPFCs. Second, there is still limited evidence from noninvasive brain stimulation studies about the imbalanced function of the left and right DLPFC in ADHD pathophysiology. While those studies with anodal left DLPFC tDCS show that increasing the activity of this region is important for cognitive deficits [30,31,32], few studies have shown that cathodal tDCS over left DLPFC is beneficial to ADHD symptoms via an indirect excitatory effect on the right DLPFC [33]. There is also one study so far that applied anodal left and cathodal right DLPFC tDCS in children with ADHD and showed improving effects in some domains of executive function and memory [30]. Applying excitatory anodal tDCS over both left and right DLPFC allows us to understand whether the upregulation of bilateral DLPFC is beneficial to cognitive deficits in ADHD.

In addition to stimulation parameters such as the target region, there are some factors related to study design that are not well-studied in tDCS studies in ADHD. One such factor is the effect of session order, i.e., whether participants received active or sham stimulation. This is especially important for the cross-over design where participants received different stimulation in a single session. This is not studied in previous works. Accordingly, the present study aims to investigate the impact of bilateral DLPFC anodal and sham tDCS on executive dysfunction in ADHD. Performance on working memory, cognitive flexibility, response inhibition, and executive control tasks that are impaired in ADHD [7,34] were examined during anodal and sham tDCS while half of the participants received the anodal session first and the rest received the sham stimulation first. Considering the documented role of anodal left DLPFC tDCS in previous studies [24] and the relevance of the right DLPFC based on limited previous works [27,33], we hypothesized an improving effect of bilateral anodal DLPFC tDCS on executive functions of children with ADHD.

## 2. Materials and Methods

### 2.1. Participants

Twenty-two right-handed, gender-controlled children diagnosed with ADHD (mean age = 8.86 ± 1.80, 11 females) were recruited from several state-based and private clinics across Mashhad, Iran. Demographic information is shown in Table 1. The required sample size was calculated a priori based on a medium critical effect size suggested for tDCS studies [35] as well as previous tDCS studies in ADHD, which included 20 subjects on average across sixteen randomized controlled trials [24]. For a within-subject repeated design with two measurements (f = 0.40, α = 0.05, power = 0.95, *n* = 23), the required sample size was 23 using the G*power software. All children were clinically interviewed based on the DSM-5 diagnostic criteria for ADHD and their parents completed the Conners’ Parent Rating Scale (CPRS) [36] and the Behavior Rating Inventory of Executive Function (BRIEF) [37]. Inclusion criteria were (1) ADHD diagnosis based on DSM-5 by a professional child psychiatrist and moderate to severe scores on the CPRS (70>); (2) 8–12 years old; (3) right-handed; (4) no current or past history of epilepsy, seizures, or head injury; and (5) no comorbidity with other neurodevelopmental disorders (e.g., oppositional defiant and conduct disorders, autism, learning disabilities). The study was performed according to the latest version of the Declaration of Helsinki ethical standards and approved by the Ethical Committee of the Shahid Beheshti University (IR.SBU.REC.1399.078). All patients’ parents were instructed about the experimental procedures and gave their informed consent before participation.

### 2.2. Executive Functions Tasks

A neuropsychological battery of four executive function tasks was used to examine working memory, cognitive flexibility, response inhibition, and executive control. Details of the tasks are described below. The order of tasks was randomized across sessions.

#### 2.2.1. Working Memory

Working memory was examined with the 1-back picture task. During the task, participants are asked to identify the picture that repeats “n” times before the onset. Here in the 1-back task, the target was any picture that was identical to the one that preceded it one trial back. The task consisted of 3 blocks, each with 30 trials, lasting around 6 min in total. The stimuli consisted of 10 different images that randomly appeared three times in each block. The mean reaction time and the number of correct responses were recorded as the outcome measures.

#### 2.2.2. Cognitive Flexibility

Cognitive flexibility was measured with a computerized version of the Wisconsin Card Sorting Test (WCST) with 64 cards. The WCST is the gold standard of executive functioning [38] and is used to measure flexibility, planning, and task-switching abilities [39,40]. The task requires participants to identify the sort criterion of a set of cards based on the “correct” versus “incorrect” feedback of the examiner. After correctly matching a card according to a stimulus feature (color, form, or number) for 10 consecutive trials, the matching feature changes. The number of categories completed, perseverative errors, and RT were the outcome measures in this task. This task takes about 7 min to complete.

#### 2.2.3. Response Inhibition

Response inhibition was measured with a computerized Go/No-Go task. In this task, participants are presented with a black plane (7 × 7 cm) appearing on a white screen in four directions: up, down, left, and right. They are instructed to press the button aligned with the plane (the Go condition), but to withhold pressing the button in case of the sound “Beep” is heard (the No-Go condition). The task consists of 50 stimuli with 75% of stimuli in the Go condition and the remaining in the No-Go condition. Outcome variables are accuracy for the Go and No-Go trials and reaction time (RT) of Go stimuli. The accuracy of No-Go is specifically considered an index of inhibitory control. This task takes about 7 min to complete.

#### 2.2.4. Executive Control

To examine executive control abilities, we used the Flanker test. In this test, a sequence of stimuli (5 arrows) is presented for 500 ms with a central stimulus (an arrow pointing to left or right) and flankers that are either congruent (pointing in the same direction as the central arrow), incongruent (pointing in the opposite direction of the central arrow), or neutral (2 horizontal lines on either side of the central arrow). The subjects are instructed to respond and decide about the direction of the central arrow as fast as possible, determining their reaction time for congruent, incongruent, and neutral stimuli. Additionally, the executive effect is among the outcome variables of interest by subtracting the congruent cue RT from incongruent cues RT. The task consists of 150 sequences of stimuli with 50 congruent, 50 incongruent, and 50 neutral conditions and takes around 6 min to complete.

### 2.3. tDCS

Direct electrical current was delivered by an electrical stimulator (NeuroStim 2, Medina Teb, Tehran, Iran) with a 9-volt battery as its source and via a pair of saline-soaked (NaCl 0.9%) sponge electrodes (electrode size of 4 × 4 cm for both electrodes). The device was equipped with 4 electrode channels enabling the delivery of bilateral stimulation with the polarity of interest. The stimulation duration was 15 min with 30 s ramping up and 30 s ramping down and a current intensity of 1.5 mA, during which participants behavioral tasks (online stimulation). For an estimate of electrical current flow, please refer to previous modeling studies [24,41]. Each participant received two sessions of active and sham stimulation. In the active condition, two anode electrodes were placed over the left and right DLPFCs, respectively (F3 and F4), according to the 10–20 International EEG System. The return electrodes were placed on the contralateral shoulders. Similar stimulation protocols (using both anodal electrodes) were already used in previous work in ADHD using slow oscillating transcranial direct current stimulation [29]. The same protocol was applied during the sham condition except that the device was turned off after 30 s of stimulation (with 30 s ramping up and 30 s ramping down) without the knowledge of the participants to generate the same sensation as in the active condition. The experimenter who applied tDCS was not blind to tDCS conditions (active vs. sham). The applied protocol is not used in other tDCS studies in ADHD to the best of our knowledge and the stimulation configuration was adapted to upregulate both left and right DLPFCs with anodal stimulation.

### 2.4. Procedure

Each subject participated in two tDCS sessions in between-subject randomized order with a 72 h interval between sessions. Half of the patients (*n* = 11) received active tDCS in the first and sham tDCS in the second session and the other half (*n* = 11) received sham tDCS in the first and active tDCS in the second session. This was to counterbalance the order of the stimulation session and to see if the order had any effect on the outcome measures. The order of tasks was randomized between subjects but the same for each subject during active and sham stimulation. During each session, participants were seated on a comfortable chair in front of a laptop with a 15.6-inch screen at a viewing distance of approximately 50 cm. After electrode placement, electrical stimulation started for 15 min. Participants were blinded to the stimulation condition. At the end of each session, participants were asked about tDCS-related side effects (e.g., itching, burning, pain) on a 5-point Likert scale [42].

### 2.5. Statistical Analysis

The experiment had a randomized, single-blinded, sham-controlled, cross-over design. All analyses were performed with the statistical package SPSS Version 28 for Windows (IBM, SPSS, Inc., Chicago, IL, USA). A series of 2 × 2 repeated-measures analyses of variance (ANOVA) was conducted on each behavioral task and their outcome measures (i.e., RT and accuracy in N-back; completed categories, perseverative errors, and RT in WCST; accuracy Go, Accuracy No-Go, and RT in Go/No-Go task; RT congruent, RT incongruent in Flanker test). In each RM ANOVA, the outcome measure of interest was entered as the dependent variable with stimulation (active vs. sham stimulation) as the within-subject factor and order (active-sham, sham-active) as the between-subject factor. The Mauchly test was performed to test for sphericity violations, and the Greenhouse–Geisser correction was applied when necessary. Conditional on significant results of the ANOVA, Fisher’s LSD post hoc tests (critical *p* < 0.05, two-tailed) were performed for post hoc analysis. One data point of two participants in the Flanker test was excluded due to technical issues during recording. A critical *p*-value of <0.05 was used for all statistical analyses.

## 3. Results

### 3.1. Data Overview

**Figure 1:**
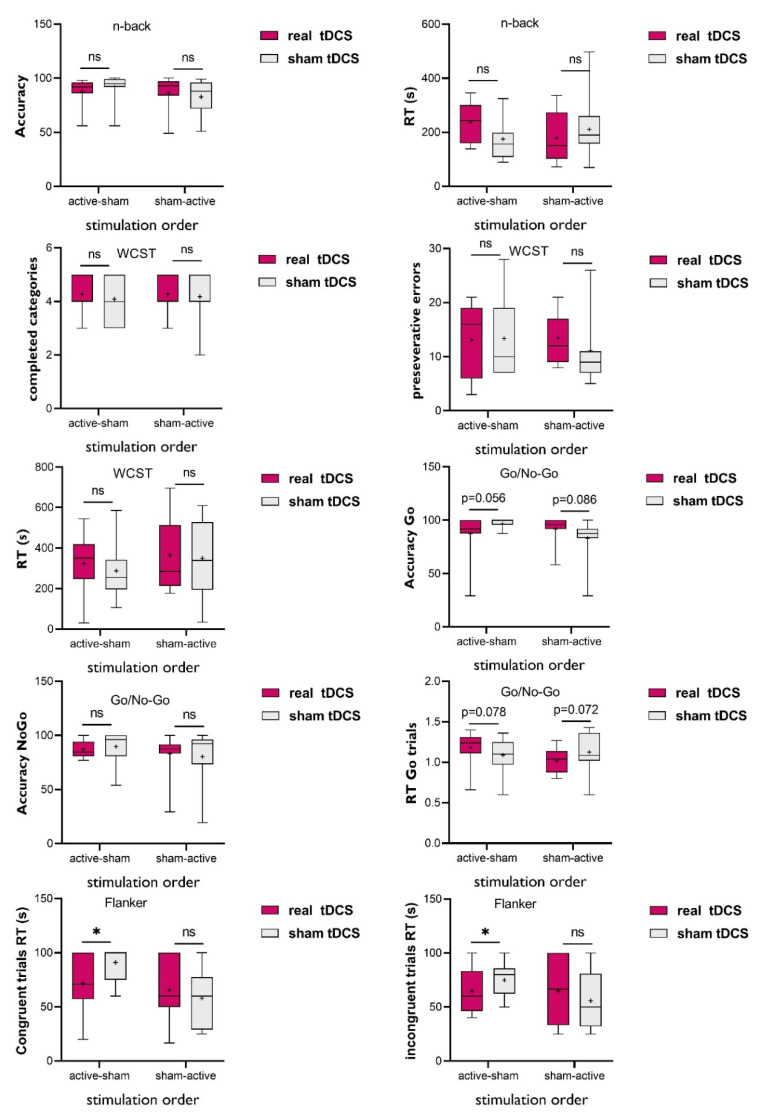
Executive functioning performance under bilateral DLPFC, anodal tDCS and sham tDCS, in children with ADHD. tDCS = transcranial direct current stimulation; RT = response time; S = second; WCST = Wisconsin Card Sorting Test; ns = non-significant. * = refers to significant difference between the real tDCS vs sham tDCS.

The demographic information of the participants is in Table 1. The stimulation was well-tolerated with a few cases of reported mild adverse effects during the stimulation; see Table 2. No significant difference was found between active and sham stimulation conditions in experiencing headache (*t*_(21)_ = 0.27, *p* = 0.789), dizziness (*t*_(21)_ = 0.43, *p* = 0.665), burning (*t*_(21)_ = 0.89, *p* = 0.383), itching (*t*_(21)_ = 0.37, *p* = 0.715), concentration problems (*t*_(21)_ = 1.00, *p* = 0.329), and sleep pressure (*t*_(21)_ = 0.64, *p* = 0.525). The data overview of dependent variables during active and sham stimulations are presented in Table 2 and Figure 1.

### 3.2. The Impact of Bilateral Anodal tDCS on Executive Functions

For the working memory task performance, measured by the n-back task, the results of ANOVA showed no significant stimulation×order interaction (*F*_(1, 20)_ = 2.07, *p* = 0.165, *η*_p_^2^ = 0.09), no significant main effect of stimulation (*F*_(1, 20)_ = 0.02, *p* = 0.880, *η*_p_^2^ = 0.01), and no significant main effect of order (*F*_(1, 20)_ = 0.97, *p* = 0.336, *η*_p_^2^ = 0.04) on accuracy scores. The same pattern of response was found for the nback RT with no significant stimulation×order interaction (*F*_(1, 20)_ = 3.18, *p* = 0.09, *η*_p_^2^ = 0.13) and main effects of stimulation (*F*_(1, 20)_ = 0.30, *p* = 0.58, *η*_p_^2^ = 0.01) and order (*F*_(1, 20)_ = 0.15, *p* = 0.703, *η*_p_^2^ = 0.01). For the cognitive flexibility measured by the WCST, again, no significant stimulation × order interaction, main effects of stimulation, and main effect of order were observed for the completed categories as the primary outcome measure (*F*_(1, 20)interaction_ = 0.04, *p* = 0.836, *η*_p_^2^ = 0.01; *F*_(1, 20)stimulation_ = 0.39, *p* = 0.535, *η*_p_^2^ = 0.02; *F*_(1, 20)order_ = 0.03, *p* = 0.862, *η*_p_^2^ = 0.01), perseverative errors (*F*_(1, 20)interaction_ = 0.61, *p* = 0.442, *η*_p_^2^ = 0.03; *F*_(1, 20)stimulation_ = 0.38, *p* = 0.545, *η*_p_^2^ = 0.02; *F*_(1, 20)order_ = 0.17, *p* = 0.684, *η*_p_^2^ = 0.01), and total RT (*F*_(1, 20)interaction_ = 0.11, *p* = 0.735, *η*_p_^2^ = 0.01; *F*_(1, 20)stimulation_ = 0.71, *p* = 0.407, *η*_p_^2^ = 0.03; *F*_(1, 20)order_ = 0.71, *p* = 0.407, *η*_p_^2^ = 0.03).

Regarding the response inhibition measured by the Go/No-Go task, the results of ANOVA showed no significant stimulation×order interaction, main effects of stimulation, and main effect of order for the accuracy of No-Go trials, which is the primary outcome measure of interest (*F*_(1, 20)interaction_ = 0.61, *p* = 0.443, *η*_p_^2^ = 0.03; *F*_(1, 20)stimulation_ = 1.05, *p* = 0.321, *η*_p_^2^ = 0.06; *F*_(1, 20)order_ = 1.03, *p* = 0.325, *η*_p_^2^ = 0.06). In Go trial accuracy and RT, a significant stimulation×order interaction (*F*_(1, 20)Accuracy_ = 5.09, *p* = 0.035, *η*_p_^2^ = 0.20; *F*_(1, 19)RT_ = 8.01, *p* = 0.011, *η*_p_^2^ = 0.29) but no main effects of stimulation (*F*_(1, 20)Accuracy_ = 0.01, *p* = 0.928, *η*_p_^2^ = 0.01; *F*_(1, 19)RT_ = 0.02, *p* = 0.878, *η*_p_^2^ = 0.01) and order (*F*_(1, 20)Accuracy_ = 0.73, *p* = 0.396, *η*_p_^2^ = 0.03; *F*_(1, 19)RT_ = 0.55, *p* = 0.465, *η*_p_^2^ = 0.02) were observed. Post hoc comparisons using *Student’s* t-test showed a trend-wise increase in Go trial accuracy during *sham* vs. active tDCS in the group that received active tDCS first (*t*_(10)_ = 1.73, *p* = 0.056). A similar trend emerged for the group that received sham tDCS first with a higher accuracy score during active tDCS vs. sham (*t*_(10)_ = 1.07, *p* = 0.086). These comparisons show a trendwise effect of session order and potentially the practice effect from the first to the second session or ceiling effect if the task was easy for the subjects. No significant difference was found for Go trials RT during active or sham tDCS for both groups (*t*_(10)_ = 1.96, *p* = 0.078; *t*_(9)_ = 2.03, *p* = 0.072).

Finally, the results of ANOVA showed a significant stimulation×order interaction effect on the RT of congruent (*F*_(1, 18)_ = 6.32, *p* = 0.022, *η*_p_^2^ = 0.27) and incongruent (*F*_(1, 18)_ = 5.39, *p* = 0.032, *η*_p_^2^ = 0.19) trials of the Flanker test. The main effects of stimulation and order were significant for neither congruent (*F*_(1, 18)_ = 1.79, *p* = 0.198, *η*_p_^2^ = 0.09; *F*_(1, 18)_ = 4.09, *p* = 0.059, *η*_p_^2^ = 0.19) nor incongruent (*F*_(1, 18)_ = 0.23, *p* = 0.633, *η*_p_^2^ = 0.01; *F*_(1, 18)_ = 0.32, *p* = 0.577, *η*_p_^2^ = 0.01) trials. No significant stimulation×order interaction or main effects of stimulation and order were found for the neutral trials. Post hoc comparisons with *Student’s* t-test showed a significant reduction in the RT of congruent (*t*_(9)_ = 3.01, *p* = 0.015) and incongruent (*t*_(9)_ = 2.38, *p* = 0.044) trials during active but not sham tDCS, only when the subjects received active stimulation first compared to when sham tDCS preceded (*t*_(9)congruent_ = 0.75, *p* = 0.472; *t*_(9)incongruent_ = 1.07, *p* = 0.312).

## 4. Discussion

In the present study, we investigated the impact of online anodal tDCS over bilateral DLPFCs (left and right DLPFC) on the executive functioning task performance (working memory, cognitive flexibility, response inhibition, and executive control) of 22 children with ADHD. We found no improving effect of anodal bilateral DLPFC tDCS on working memory, cognitive flexibility, and response inhibition. A partial improving effect of this tDCS montage was, however, found on performance reaction time in the Flanker test for both congruent and incongruent trials. This effect, however, was dependent on stimulation order and observed when the subjects received the active tDCS in the first session.

Mixed results about the efficacy of tDCS in ADHD, related to targeting heterogeneous cortical regions, the use of suboptimal protocols, and the lack of systematic investigation of stimulation parameters (e.g., intensity, polarity, target regions), warrant more research in this field [20]. The DLPFC is the most targeted cortical region in tDCS studies of ADHD, with 64.71 of RCTs published by early 2022 [20]. There is, however, limited knowledge about the contribution of the DLPFC laterality (i.e., only left DLPFC, only right DLPFC, left and right DLPFC) and stimulation polarity (i.e., only anodal, only cathodal, anodal–cathodal). Previous studies have already addressed some of these combinations, including anodal left–cathodal right DLPFC [30], anodal/cathodal left DLPFC in combination with the right orbitofrontal cortex [30,43], anodal right DLPFC (with extracranial reference electrode) [27], and anodal left DLPFC (with reference electrode over the vertex) [31,32]. No study so far has investigated the impact of upregulatory anodal tDCS over both left and right DLPFC, which was the primary rationale of our study.

The null effect of bilateral DLPFC anodal tDCS needs to be interpreted with previous works and has several implications. The protocol applied in this study was aimed to increase activity in both left and right DLPFCs by applying anodal stimulation, which increased cortical excitability [12]. One possible explanation here is that there is a compensatory pattern of activity in the brain of individuals with ADHD which does not benefit from the upregulation of both hemispheres. For example, some tDCS studies on ADHD showed that the cathodal stimulation of the left DLPFC might lead to an increase in activity in the right DLPFC via transcallosal connections [33]. Even if there is not a compensatory functional mechanism, it is clear that not all brain regions in ADHD benefit from external upregulatory intervention. Neuroimaging studies have shown that different cortical and subcortical areas in the brain are either hypoactive or hyperactive compared to normal peers [44]. The upregulation of both DLPFC with anodal tDCS and the relatively large size electrodes, which induce a diffuse electrical field in broad regions, might not be the best possible intervention, then. Another explanation is the possibility of counteracting each DLPFC stimulation due to the possible inhibitory links between the left and right DLPFC, as shown in some studies conducted on motor areas [45]. Some studies have also shown that the two hemispheres have distinct DLPFC and M1 cortico-cortical connectivity at rest [46].

It is also important to consider that the current design does not allow us to infer the contribution of DLPFCs in other configurations (i.e., up/downregulation of right and/or left DLPFC alone and concurrently with other regions), as we did not examine other protocols in this study. The results can only indicate that the upregulation of left and right DLPFC with online anodal tDCS does not affect behavioral performance in EFs tasks. Here, also, the results at best show that a single session of bilateral anodal tDCS does not affect the performance of executive functioning in children with ADHD. It is possible that repeated tDCS sessions with the same protocol or HD tDCS [47] would result in improving executive deficits, as shown in previous tDCS studies in ADHD [31,48] or other psychiatric disorders marked by executive dysfunction [49,50,51,52,53]. Moreover, and related to this, our results are limited to behavioral performance and cannot deliver information about the physiological and neural effects of this protocol on the parameters of brain physiology. Last but not least, there are other external factors with a huge impact on tDCS-induced plasticity and cognitive function such as the time of the day that stimulation is delivered [54] and sleep [55]. These factors are also impaired in the ADHD population [56] and need to be controlled in order to see the real effects of the stimulation.

Future studies need to replicate this protocol using both behavioral and neurophysiological measures. For example, the use of neuroimaging methods (e.g., EEG, fMRI, TMS-EEG) can uncover the effects of this protocol on the developing brain of children with ADHD more comprehensively. Besides, our study had several limitations to be addressed in future works, including a single-blind design, no use of neurophysiological measures, and also a small sample size. The protocol we used in this study is novel. Future works need to replicate and optimize it if needed.

## Figures and Tables

**Table 1:** Demographic information.

Variable	Category	Value
Age—Mean (SD)		8.86 (1.80)
Sex—Male (female)		
school year (n)	Class 1 (7 years old)	8
	Class 2 (8 years old)	3
	Class 3 (9 years old)	3
	Class 4 (10 years old)	1
	Class 5 (11–12 years old)	7
CPRS—Mean (SD)	Overall score	73.40 (13.29)
BRIEF—Mean (SD)	Inhibit	15.81 (4.10)
	Shift	10.50 (3.81)
	Emotional Control	10.77 (4.89)
	Initiate	9.13 (3.15)
	Working Memory	12.27 (3.94)
	Plan/Organize	16.36 (5.63)
	Organization of Materials	8.63 (3.25)
	Monitor	10.18 (3.45)

Note: SD = Standard Deviation; CPRS = The Conners’ Parent Rating Scale; BRIEF = Behavior Rating Inventory of Executive Function.

**Table 2:** Means and SDs of the reported side effects during tDCS, working memory, cognitive flexibility, response inhibition, and executive control task performance.

Task	Outcome Measures	Group 1 (Active-Sham)		Group 2 (Sham-Active)	
		Active tDCS M (SD)	Sham tDCS M (SD)	Active tDCS M (SD)	Sham tDCS M (SD)
**tDCS side effects**	Headache	0.36 (0.67)	0.18 (0.40)	0.90 (0.30)	0.36 (0.92)
	Dizziness	0.90 (0.30)	0.09 (0.30)	0.18 (0.60)	0.90 (0.30)
	Burning sensation	0.45 (0.68)	0.27 (0.64)	0.18 (0.40)	0.72 (1.27)
	Itching	0.63 (1.20)	0.90 (1.37)	0.54 (0.68)	0.36 (0.67)
	Concentration problems	0.00 (0.00)	0.00 (0.00)	0.00 (0.00)	0.18 (0.60)
	Sleep pressure	0.09 (0.30)	0.27 (0.90)	0.45 (1.21)	0.00 (0.00)
**Working memory (n-back)**	Accuracy	87.72 (12.54)	92.27 (12.41)	86.45 (17.29)	82.72 (15.23)
	RT *	236.44 (73.05)	174.75 (81.38)	178.40 (86.33)	211.03 (115.43)
**Cognitive flexibility (WCST)**	Completed categories	4.27 (0.64)	4.09 (0.83)	4.27 (0.64)	4.18 (0.98)
	Perseverative error	13.09 (6.90)	13.36 (7.17)	13.45 (4.63)	11.18 (6.64)
	RT *	323.89(137.92)	287.88(131.74)	365.03(177.27)	349.81 (184.13)
**response inhibition (Go/NoGo)**	Accuracy No Go	87.17 (7.69)	86.32 (17.42)	80.41 (25)	73.93 (29.02)
	Accuracy Go	87.12 (20.53)	96.59 (4.86)	91.66 (11.87)	82.95 (19.40)
	RT * Go trials	1.18 (0.20)	1.08 (0.21)	1.01 (0.15)	1.12 (0.25)
**Executive control (Flanker test)**	RT * congruent	71.59 (25.25)	90.16 (16.22)	63.67 (27.44)	58.01 (25.98)
	RT * incongruent	64.94 (21.17)	77.72 (14.08)	66.47 (30.29)	55.69 (25.66)
	RT * neutral	74.49 (30.57)	77.87 (22.31)	70.47 27.99	52.28 (28.65)

Note: Active tDCS = M = means; SD = Standard Deviation; tDCS = transcranial direct current stimulation; RT = response time; WCST = Wisconsin Card Sorting Test; * = RT in seconds and in WSCT and working memory refer to the total time taken by the subject to finish the task.

## Data Availability

The data generated from this study are available upon request.
